# Effects of Maternal Nutrition, Resource Use and Multi-Predator Risk on Neonatal White-Tailed Deer Survival

**DOI:** 10.1371/journal.pone.0100841

**Published:** 2014-06-26

**Authors:** Jared F. Duquette, Jerrold L. Belant, Nathan J. Svoboda, Dean E. Beyer, Patrick E. Lederle

**Affiliations:** 1 Carnivore Ecology Laboratory, Forest and Wildlife Research Center, Mississippi State University, Mississippi State, Mississippi, United States of America; 2 Michigan Department of Natural Resources, Wildlife Division, Marquette, Michigan, United States of America; 3 Michigan Department of Natural Resources, Wildlife Division, Lansing, Michigan, United States of America; University of Illinois at Urbana-Champaign, United States of America

## Abstract

Growth of ungulate populations is typically most sensitive to survival of neonates, which in turn is influenced by maternal nutritional condition and trade-offs in resource selection and avoidance of predators. We assessed whether resource use, multi-predator risk, maternal nutritional effects, hiding cover, or interactions among these variables best explained variation in daily survival of free-ranging neonatal white-tailed deer (*Odocoileus virginianus*) during their post-partum period (14 May–31 Aug) in Michigan, USA. We used Cox proportional hazards mixed-effects models to assess survival related to covariates of resource use, composite predation risk of 4 mammalian predators, fawn body mass at birth, winter weather, and vegetation growth phenology. Predation, particularly from coyotes (*Canis latrans*), was the leading cause of mortality; however, an additive model of non-ideal resource use and maternal nutritional effects explained 71% of the variation in survival. This relationship suggested that dams selected areas where fawns had poor resources, while greater predation in these areas led to additive mortalities beyond those related to resource use alone. Also, maternal nutritional effects suggested that severe winters resulted in dams producing smaller fawns, which decreased their likelihood of survival. Fawn resource use appeared to reflect dam avoidance of lowland forests with poor forage and greater use by wolves (*C. lupus*), their primary predator. While this strategy led to greater fawn mortality, particularly by coyotes, it likely promoted the life-long reproductive success of dams because many reached late-age (>10 years old) and could have produced multiple generations of fawns. Studies often link resource selection and survival of ungulates, but our results suggested that multiple factors can mediate that relationship, including multi-predator risk. We emphasize the importance of identifying interactions among biological and environmental factors when assessing survival of ungulates.

## Introduction

Survival of neonates typically has greater influence on ungulate population growth than other vital rates due to wide temporal variation [1], [2], [3] and greater susceptibility of neonates to limiting factors [4], [5], [6]. Neonatal mortality in ungulates is sensitive to variation in numerous biological and environmental factors [6], but especially body condition at birth [2], [3], [7], limiting resources (e.g., hiding vegetation; [8], [9]), and predation [10]. These factors are often interrelated and affect survival directly through predation or indirectly through resource use and maternal nutritional effects, which can influence neonate body growth and consequently survival [11], [12], [13]. Additionally, weather conditions can mediate the degree to which these factors, including predation [7], [14], [15], affect survival. Hence, identifying limiting factors that cause variation in survival of neonates presents a major consideration for understanding ungulate population growth and management [9].

Survival rates of neonate ungulates often decrease the greatest during the first three months of life (e.g., [16]), when they are maternally dependent. Neonate dependency on dams can influence their survival through variation in the nutritional condition [7], [17] and resource use of dams [18], [19], which can make neonates vulnerable to predation [20], [21], [22], [23], [24]. Although predation directly limits survival, spatiotemporal variation in predation risk can indirectly mediate resource selection of parturient females and survival of neonates [8], [25]. During spring-summer, parturient females must use behavioral trade-offs to acquire forage to meet nutritional demands [11] while reducing predator detection of neonates [8], [10], [26]. For example, parturient females may reduce their detection by predators [27] by modifying their vegetation and space use (e.g., parturition areas; [8], [10], [28]), increasing vigilance [29], [30], [31], [32], or using refuge cover [33], [34], [35]. However, annual variation in the vegetation available for forage and neonate hiding cover can influence the magnitude of the trade-off between nutritional gain and hiding fawns or avoiding predators for dams. Therefore, neonate survival is an appropriate metric to assess the influence of trade-offs in resource use and predation risk on population growth.

Although a single predator species can directly limit survival of neonates (e.g., [36]), multiple predators can have cumulative negative effects on survival [37] and population growth [38]. Ungulates using multi-predator landscapes face challenges of variation in species-specific predator hunting strategies [39], [40] and temporal efficacy of predation [16], [41]. Variation in predation risk can mediate ungulate selection or avoidance of particular resources [15], [37], and can have underlying additive or interactive effects on survival beyond that of resource selection alone [42]. Behavioral trade-offs in resource selection and species-specific predator avoidance are therefore essential to survival of white-tailed deer (*Odocoileus virginianus*), but may be confounded by avoidance of multi-species predation risk. For example, parturient females may select specific resources to avoid wolves, which can expose neonates to greater risk from other predators [34]. Assessing the interactive effects of resource use of neonates, predator risk, and weather conditions can help elucidate variation in neonatal ungulate survival.

White-tailed deer fawns are maternally dependent for about 60 days post-parturition [43], meaning that their resource use and predation risk are dictated primarily by dams [44]. To enhance nutritional intake and reduce the detection of fawns by predators, parturient females isolate themselves from conspecifics and adjust their space use to areas with vegetation that provides hiding cover for fawns [10], [45], [46]. Additionally, white-tailed deer have been shown to select areas that reduce spatial overlap with primary predators such as gray wolves [47], [48], though predators may actively search areas of greater probability of encountering fawns [49]. However, if forage is limiting, parturient females may choose to increase nutritional intake rather than avoid predation risk [24]. Therefore, the hiding strategy of fawns (e.g., bedding in dense vegetation; [46]) is essential to reducing predator detection.

Studies assessing effects of predation risk on neonatal ungulate survival have typically been limited to single predator species [8], [10], [50], which may not fully reflect ungulate life history strategies within multi-predator systems. We assessed daily survival of white-tailed deer fawns in relation to resource use, concomitant cumulative resource selection of bobcats (*Lynx rufus*), American black bears (*Ursus americanus*), coyotes (*C. latrans*), and gray wolves, birth body mass, vegetation growth, and an index of winter weather severity during the fawn post-partum period (May–Aug). We did not have behavioral data on parturient females associated with fawns in our study, but assumed that maternal behaviors generally dictated the resource use and predator avoidance of fawns [33], [44].

Similar to [51], we made 6 predictions describing different resource use and predation risk relationships to survival outcomes in a landscape with multi-predator risk. “Ideal resource use” prediction assumed that variation in fawn survival was influenced by ideal free resource selection of dams [52], [53], whereby a decrease in ideal resource use would increase the mortality hazard, irrespective of variation in predation risk. “Predation risk” prediction assumed that variation in fawn survival was influenced by variation in predation risk, whereby an increase in predation would increase the mortality hazard, irrespective of variation in resource use. “Non-ideal resource use” prediction assumed that a decrease in ideal resource use would increase the mortality hazard with additive predation risk within those resources further increasing the mortality hazard [42]. The “non-ideal resource use” prediction also assumes that dam interpretation of habitat quality is imperfect and their resource selection is not mediated by variation in predation risk [42], [51]. “Ecological trap” prediction assumed similar resource use and predation risk relationships as “non-ideal resource use”, but assumed that resource use was mediated by the variation in predation risk perceived by dams leading to preference for poor-quality sink habitats [54]. “Maternal effects” assumed that annual variation in survival is influenced by birth mass and winter weather severity or their interaction [7], irrespective of other variables. “Hiding cover” assumed that variation in survival was influenced by spring vegetation growth phenology [26], irrespective of other variables.

## Materials and Methods

### Ethics statement

Ethics of all capture and handling procedures were approved by the Mississippi State University Institutional Animal Care and Use Committee (#09-004). Also, animal capture and handling procedures followed guidelines established by the American Veterinary Medical Association and the American Society of Mammalogists [55]. Field studies did not involve endangered or protected species. We conducted most field activities on land owned by the Michigan Department of Natural Resources that granted access for our study, but several private land parcels were accessed with landowner permission. Data used in analyses can be obtained from the Dryad (http://datadryad.org/) repository.

### Study area

**Figure 1:**
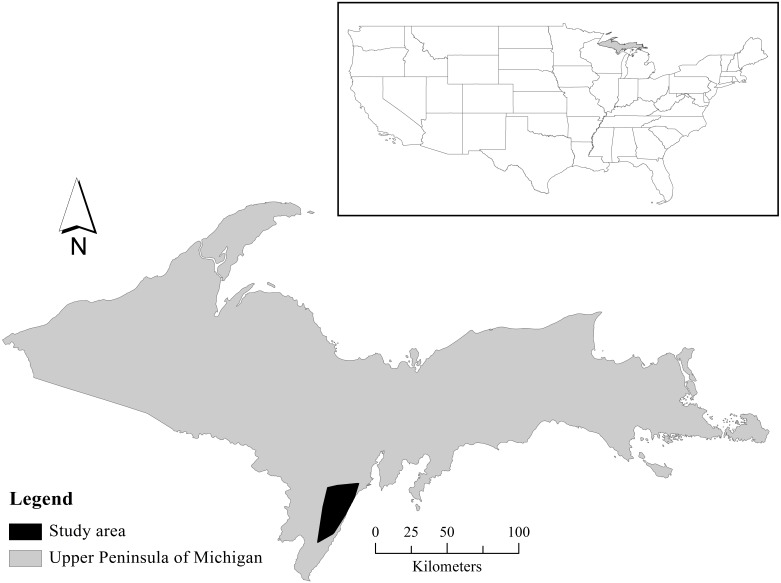
Location (black polygon) of white-tailed deer (*Odocoileus virginianus*) resource use and predation risk study, Upper Peninsula of Michigan, USA, 2009–2011.

We conducted our study in the south-central Upper Peninsula of Michigan (45°43′47″ N, 87°4′48″ W; Fig. 1). Mean elevation is 185 m above sea level and topography is flat. Lowland forests generally occurred away from roads and were mainly composed of eastern white cedar (*Thuja occidentalis*), eastern hemlock (*Tsuga canadensis*), and balsam fir (*Abies balsamea*) with areas of alder shrubs (*Alnus spp*.). Upland forests included pine (*Pinus spp.*), aspen (*Populus spp.*), maple (*Acer spp.*), and birch (*Betula spp.*) trees. Grasses and shrubs were mixed and uncommon in the study area. The study area was interspersed with 10% cropland (mainly corn [*Zea spp.*] and soybeans [*Glycine spp.*]) and 3% pasture, mainly in the western half of the area. Developed area included low density (0.09 km/km^2^) residential and recreational camps. Roads were predominantly paved, but several were gravel or soil; overall road density was 1.68 km/km^2^. Permanent water (e.g., rivers and lake shoreline) density was 1.17 km/km^2^. From 2009 through 2011, mean monthly temperature ranged from 10.4°C in May to 19.0°C in August using a site-specific weather station sensor (model 107-L, Campbell Scientific Inc., Utah, USA). Annual density of adult and fawn white-tailed deer was 3.7–3.9/km^2^ and 0.6–1.3/km^2^, respectively, based on remote camera surveys [1]. Bobcat density was 0.03/km^2^ [56] and black bear density was 0.14–0.19/km^2^ based on hair snare surveys (Belant, J.L., unpublished data). Coyote density was 0.32–0.37/km^2^ based on howl elicitation surveys [57] and wolf density was 0.012/km^2^ based on winter track surveys (Petroelje, T.R. unpublished data).

### Fawn capture and monitoring

We captured 129 neonatal fawns (estimated ≤15 days old; 69 males, 58 females, 2 unknown) opportunistically (*n* = 100) or with vaginal implant transmitter searches (*n* = 29; [58]) of radio-collared adult females from May to July 2009–2011. These methods, particularly vaginal implant searches, allowed us to capture fawns throughout the study area and minimize bias of captures near roads. We weighed fawns to the nearest 0.01 kg using a spring scale (model#80020; Pesola; Kapuskasing, Ontario, Canada) and then fit each with an expandable radio collar (model 4210, Advanced Telemetry Systems Inc., Minnesota, USA). We attached 2 ear tags (model agpf#1, Allflex, Texas, USA), identified sex, estimated birth date and age based on new hoof growth [7], and then released fawns at their site of capture. We estimated fawn birth body mass by subtracting the average daily mass gain for northern, newborn white-tailed fawns (0.2 kg) from the capture mass [7].

Each year, we relocated radio-collared fawns on a diel schedule up to 5 times/week from birth to 31 Aug using truck-mounted 3 or 4 element Yagi antenna or aerial radiotelemetry using a 2 element antenna. Seventy-six percent of relocations were obtained during day hours (07∶00–18∶59) and 24% were obtained during night hours (19∶00–06∶59). We estimated fawn locations from the ground using ≥3 bearings collected within 20 min [59] and Location of a Signal 4.0 software (Ecological Software Solutions LLC, Hegymagas, Hungary). We aerially estimated fawn locations by passing over each individual radio signal ≥2 times at low altitude (i.e., ≤244 m) within 10 min and recording the location where we heard the loudest signal. We assessed telemetry error for personnel conducting ground and aerial telemetry by placing 5 radio collars in forested or non-forested (e.g., pasture) vegetation, calculated mean ellipse error (2115 m^2^) from the known location of radio collars, and discarded locations with error ellipses larger than the mean error.

After detecting a radio collar mortality signal, we investigated mortality sites within 8 hr and assessed whether the signal was due to fawn mortality or other causes (e.g., slipped radio collar). When mortalities occurred, we searched sites generally within 200 m of the radio collar and expanded searches if evidence of mortality was found within this search zone. We attributed mortalities to specific predators based on predation characteristics, carcass wounds, and site characteristics, which we compared to published descriptions [60], [61], [62], [63].

### Resource use

We used third order selection analysis [64] with design 3 [65] to estimate resource use probability of fawns within their respective individual area of use. We defined resource use as fawn radiolocations (*N* = 2713; 2–56 locations/fawn) from birth to censor date, or 31 Aug, and defined resource availability as a point randomly generated within 415 m from each radiolocation using Geospatial Modelling Environment (Version 0.7.1.0; [66]), based on cumulative mean step length between radiolocations of each fawn. We then used ArcGIS 10.0 [67] to buffer radiolocations and random points with mean ellipse error (radius = 26 m) to account for telemetry error in selection analysis and provide analogous sampling methods. We estimated availability using random point buffers because most fawns died before ≥30 radiolocations could be obtained to estimate home range ellipses [59]. We obtained vegetation data using 2006 National Landcover Data (30-m resolution; [68]) that we reclassified from 15 original vegetation classes to 8 (Table 1). We used ArcGIS to clip radiolocation and random point buffers from landcover data and recorded the proportion of each vegetation class within each buffer.

**Table 1:** Resource metrics used to assess resource use of white-tailed deer (*Odocoileus virginianus*) fawns, Upper Peninsula of Michigan, USA, 2009–2011.

Metric	Definition
Lowland forest (%)	Forest with moist soil, periodically saturated with water and >20% of total vegetation cover
Deciduous forest (%)	Forest with >75% deciduous trees that are >5 m tall and >20% of total vegetation cover
Coniferous forest (%)	Forest with >75% coniferous trees that are >5 m tall and >20% of total vegetation cover
Mixed forest (%)	Forest with a mix of deciduous and coniferous trees that individually comprise <75% of total tree cover
Grass/shrub (%)	Vegetation >80% graminoid or herbaceous, or trees or shrubs <5 m tall
Pasture (%)	Grasses, legumes, or grass-legume mixtures for livestock grazing or production of seed or hay crop
Cropland (%)	Fields used for row crop (e.g., soybearn or corn) production, including orchards and land actively tilled
Wetland (%)	Soil is periodically saturated with or covered with water and is >80% perennial herbaceous vegetation
Distance to road (m)	Measure of the distance from a point of interest (e.g., deer radiolocation) to theedge of the nearest secondary or primary road, including intensively used motorized-vehicle trails

We developed primary recreational vehicle trail data by traversing trails with global positioning system units and converted these data to line shapefiles using ArcGIS 10.0 [67]. We obtained road Topologically Integrated Geographic Encoding and Referencing system files [69] and merged primary recreational vehicle trails to roads because roads and trails can affect white-tailed deer behavior (e.g., predator risk avoidance, [24]). We estimated the distance to the nearest road by conducting a spatial join between the nearest road to each radiolocation or random point.

We standardized all candidate resource metrics to z-scores and centered scores to provide equal weight in multiple regression analyses [70]. We used variance inflation factor (VIF) analysis to assess multicollinearity among candidate resource metrics, with collinearity considered ≥7 [71]; no metrics were correlated (VIF = 1.12–3.59). We used package *lme4* [72] in program R to produce a generalized linear mixed-effects model of each resource using a maximum likelihood estimator and binomial distribution. We used radiolocations (1) and random points (0) as the binomial response variable and 8 vegetation classes, vegetation growth, and distance to road as fixed effects with individual fawn and year as random effects on the intercept to account for variation among fawns and years [73]. We verified fit of each model by examining standardized versus fitted residual plots. We then created an additive model with all individually significant (α = 0.05) resources to estimate probability of resource use of fawns. We estimated prediction error for each model using *k*-fold cross validation [74] by partitioning our data into 5 folds and training each model iteratively using 4 of the 5 data sets using logistic regression. We based validation on the remaining testing set.

We used the Geospatial Modelling Environment (Version 0.7.1.0; [66]) to create a grid of non-overlapping square cells (2115 m^2/^cell; mean ellipse error) across the study area. We then converted raster cells to centroid points and extracted landcover values of points to program R 3.0 [75] and summarized the proportion of each landcover class in each grid cell. We estimated the geometric centroid of each sampling grid cell and distance from each centroid to nearest road. We used standardized coefficients from the additive generalized linear resource use model to spatially derive a relative value of resource suitability (*w*; [76]) for each cell for fawns:
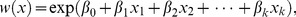
(1)


where *β*_k_ are the coefficients of the variables (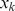). Summed coefficients could be a negative value or a value greater than 1; therefore, we used a linear stretch [77] to constrain fawn resource suitability (*w*) of each cell between 0 and 1:
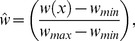
(2)where *w_min_* and *w_max_* represent the least and greatest resource use values, respectively. As standardized values (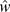) approach 1, the grid cell has a relatively greater likelihood of being selected by fawns. We appended resource suitability values to corresponding sampling grid cells shapefile and plotted the layer using ArcGIS.

### Winter severity and hiding cover

To estimate winter severity, we deployed a weather station that measured daily mean snow depth (cm), mean wind speed (kph), rainfall (cm), and minimum ambient temperature (C) near the center of the study area in a representative mixed coniferous and deciduous upland forest. We estimated mean daily winter severity from 1 Jan to 31 Mar 2009–2011 by averaging the sum of snow depth, wind speed, and rainfall and subtracting that value from daily minimum temperature. We then summed daily values for the 3-month period each year. Values were centered on 0, with increasing values indicating greater winter severity. We developed this index because of limited variation in snow depths and temperatures that were predominantly below levels used by other indexes (e.g., [78]).

We used Normalized Difference Vegetation Index (250 m resolution; [79]) data as a metric of vegetation growth that could relate to green vegetation available for fawn hiding cover [11] during spring. We obtained 2009–2011 growth values using the available 16 day composite data period closest to 1 Jun, when peak fawn parturition occurred during these years (Duquette, J.F., unpublished data). We used ArcGIS 10.0 [67] to clip radiolocation and random point buffers from vegetation growth data and estimated the mean vegetation within each buffer. There were 19,883 cells of vegetation growth data within the study area.

### Predation risk

We used previously developed spatial models estimating the probability of predator resource selection in our study area (Svoboda, N.J., unpublished data) as surrogates of predation risk [24], including bobcat, black bear, coyote, and gray wolf. Predator models were developed using resource selection functions from a total of 23,135 to 101,874 global positioning system locations of 7 bobcats, 29 black bears, 21 coyotes, and 8 gray wolves from 25 May to 31 Aug 2009–2011. These models were developed using the same grid configuration and cell size used for fawns. We estimated species-specific predation risk by clipping each predator resource selection map to the same grid configuration used for fawns and appended these values to match fawn resource suitability grid cells. Similar to resource use, we used ArcGIS to clip fawn radiolocation buffers (2115 m^2^) from predation risk grid cells, from which we estimated the mean predation risk of each predator within each buffer, which we summed to estimate composite predation risk. We estimated composite predation risk because each predator was attributed to a proportion of fawn mortalities in survival risk sets and may have influenced resource use of fawns.

### Survival analysis

We used Cox-proportional hazards mixed-effects survival models in package *coxme* [80] in R 3.0 [75] to assess whether resource use, predator risk, birth body mass, winter severity, and hiding cover or additive models of these covariates best influenced fawn survival and to account for variation in fawns among years. These models are semi-parametric regression models commonly used for survival data (e.g., [25]) and estimate proportional changes in the baseline survival hazard over time and relative differences in the hazard in relation to model covariates [81]. We used the birth date of each fawn as the start time and date of censor, death, or 31 Aug as the stop time for models. Plots of daily fawn stop times and year showed clumped distribution within individual years, therefore we used individual fawn and year as random effects in all models. We estimated percent integrated deviance explained by subtracting the log-likelihood of an individual covariate model from the log-likelihood of the null model [74] and ranked models by deviance explained.

### Spatially-predictive mortality

We used the Geospatial Modelling Environment (Version 0.7.1.0; [66]) to create a grid of non-overlapping square cells (2115 m^2^/cell; mean telemetry error) across the landscape that was available to fawns. We spatially extrapolated survival coefficients from individual resource use models by estimating survival rates to the end of each period (*S*[*te*]) as a function of the resources or predation risk of each pixel according to:
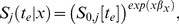
(3)where 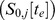 is the baseline cumulative survival probability per year to 31 Aug, with different baseline estimates according to year, *j*, [80]. We then used a linear stretch (equation 2; [77]) to scale relative probability of fawn mortality between 0 and 1, with a greater likelihood of fawn mortality as standardized grid cell values approach 1. We imported the resulting values into ArcGIS and plotted values within the study area grid.

## Results

### Fawn capture and monitoring

Mean fawn birth body mass was 2.47 kg (SD = 0.78, *n* = 42) in 2009, 4.16 kg (SD = 1.62, *n* = 35) in 2010, and 4.11 kg (SD = 0.93, *n* = 47) in 2011. We obtained 2,713 (median = 23, SD = 12.7) radiolocations from 129 fawns. There were 23 (17 predations) fawn mortalities in 2009, 17 (12 predations) in 2010, and 25 (20 predations) in 2011. Predation was primarily attributed to coyotes (47%), followed by bobcats (23%), black bears (8%), and wolves (8%). Causes of remaining mortalities (14%) were unknown or other predators.

### Resource use and winter severity

We evaluated 8 models based on individual covariates of resource use (Table 2) and retained significant models including lowland, deciduous, and coniferous forest, pasture, wetland, and distance to nearest road. An additive model of significant resources (*k*-fold prediction error = 0.13) suggested that fawns avoided lowland forest (*β* = −0.157, SE = 0.039, *P*<0.001), deciduous forest (*β* = −0.082, SE = 0.035, *P* = 0.021), coniferous forest (*β* = −0.130, SE = 0.032, *P*<0.001), and wetland (*β* = −0.067, SE = 0.029, *P* = 0.022). Also, fawns used areas closer to roads (*β* = −0.603, SE = 0.035, *P*<0.001), but pasture (*β* = −0.021, SE = 0.032, *P* = 0.501) was used in proportion to availability. Mean vegetation growth was 0.015 (SD = 0.929, *n* = 19882) in 2009, 0.592 (SD = 0.796, *n* = 19882) in 2010, and −0.607 (SD = 0.885, *n* = 19882) in 2011. Winter severity was greatest during 2009 (455.9), followed by 2011 (242.5) and 2010 (−12.7).

**Table 2:** Generalized linear mixed-effect models assessing third order resource selection of white-tailed deer fawns (≤14 weeks of age; *Odocoileus virginianus*; *n* = 129) during the post-partum period (14 May–31 Aug), Upper Peninsula of Michigan, USA, 2009–2011.

Parameters	Coefficient	Standard error	*z*-value	*P*-value	Prediction error
Lowland forest (%)	−0.207	0.027	−7.589	<0.001	0.16
Deciduous forest (%)	0.055	0.028	2.021	0.043	0.25
Coniferous forest (%)	−0.110	0.028	−3.966	<0.001	0.25
Mixed forest (%)	0.008	0.027	0.288	0.774	0.25
Grass/shrub (%)	0.006	0.027	0.215	0.830	0.25
Pasture (%)	0.082	0.027	2.978	0.003	0.25
Cropland (%)	−0.023	0.027	−0.847	0.397	0.25
Wetland (%)	−0.067	0.029	−2.299	0.022	0.25
Distance to road (m)	−0.649	0.034	−18.865	<0.001	0.14

Models used radiolocations (1; *n* = 2713) and random points (0) as the binomial response variable and individual resources were used as a fixed effect with individual fawn and year as random effects on the intercept. Model prediction error was estimated using *k*-fold cross validation using 5 folds.

### Survival models

**Figure 2:**
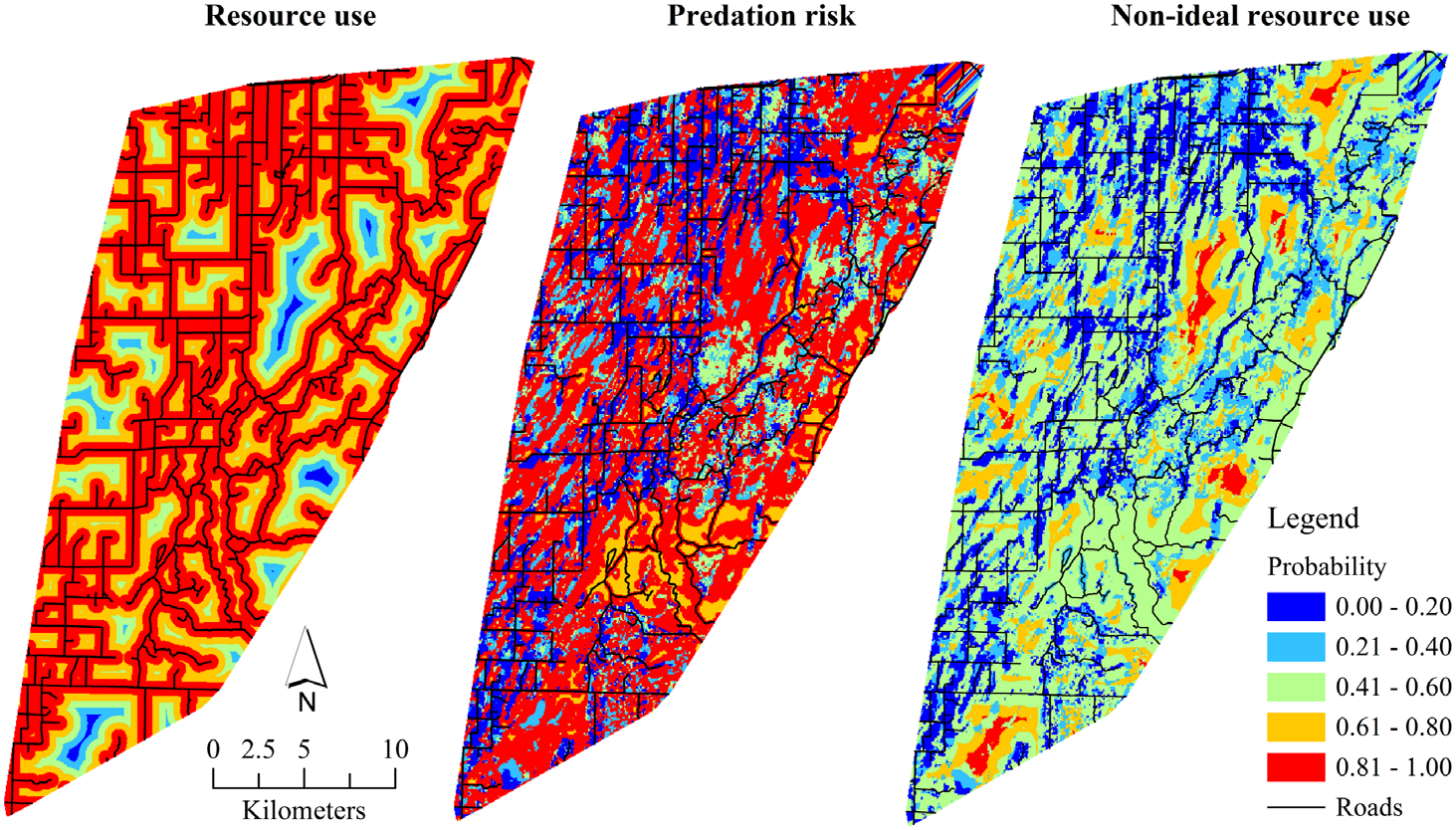
Spatially-predicted probability of resource use, composite predation risk, and non-ideal resource use for white-tailed deer fawns (*Odocoileus virginianus*; ≤14 weeks old; *n* = 129) captured as neonates during the post-partum period (25 May–31 August), Upper Peninsula of Michigan, USA, 2009–2011. Composite predation risk was estimated from the summed probability of resource selection of bobcats (*Lynx rufus*), American black bears (*Ursus americanus*), coyotes (*Canis latrans*), and gray wolves (*C. lupus*).

We evaluated 12 models related to our predictions of resource use, predation risk, maternal effects, and hiding cover on fawn survival (Table 3). An additive model including non-ideal resource use and maternal effect variables explained the greatest amount of variation (71%) in fawn survival. Similar models including additive variables of resource use, predation risk, and maternal effects explained less variation (64.05–47.19%) in fawn survival. However, maternal effects appeared to explain most of the variation in fawn survival, as deviance explained decreased substantially for models not including these factors, particularly body mass at birth. The spatially extrapolated model of resource use of fawns showed that probability of resource use greatly increased closer to roads and decreased toward interior lowland forests (Fig. 2). The predation risk model showed broad variation in risk across the study area, but increased risk did appear more spatially homogenous where roads were less dense and lowland forest was present. The extrapolated non-ideal resource use model with fawn resource use and predation risk highlighted that areas of decreased resource use suitability and increased predation risk had greater probability of mortality.

**Table 3:** Cox-proportional hazards mixed-effects models assessing the effects of resource use, predation risk, birth body mass, winter severity, and vegetation hiding cover on the daily survival of white-tailed deer fawns (≤14 weeks of age; *Odocoileus virginianus*; *n* = 129) during the post-partum period (14 May–31 Aug), Upper Peninsula of Michigan, USA, 2009–2011.

Model	Estimate	SE	*P*-value	*df*	Hazard ratio	Devianceexplained (%)	Log-likelihood χ^2^	χ^2^ *P*-value
**Non-ideal resource use +** **Maternal effects**				2695		70.78	141.56	<0.001
Resource use	−0.561	0.194	<0.001		0.571			
Predation risk	0.165	0.211	0.430		1.179			
Birth body mass	−2.784	0.539	<0.001		0.062			
Winter severity index	0.146	0.501	0.770		1.157			
Birth body mass * Winterseverity index	−0.8112	0.330	0.014		0.444			
**Maternal effects**				2695		64.05	128.10	<0.001
Birth body mass	−2.685	0.518	<0.001		0.068			
Winter severity index	0.177	0.588	<0.001		1.194			
Birth body mass * Winterseverity index	−0.879	0.333	<0.001		0.415			
**Ecological trap +** **Maternal effects**				2695		60.42	120.85	<0.001
Resource use	−0.380	0.126	0.002		0.684			
Predation risk	0.298	0.178	0.090		1.347			
Resource use * Predation risk	0.326	0.118	0.006		1.386			
Birth body mass	−1.615	0.254	<0.001		0.199			
Winter severity index	−0.132	0.264	0.620		0.876			
Birth body mass * Winterseverity index	−0.670	0.202	<0.001		0.512			
**Body mass**	−2.639	0.490	<0.001	2695	0.072	60.12	120.24	<0.001
**Ecological trap +** **Hiding cover**				2713		47.96	95.93	<0.001
Resource use	−0.608	0.215	0.005		0.544			
Predation risk	0.235	0.218	0.280		1.264			
Resource use * Predation risk	−0.160	0.273	0.560		0.852			
Vegetation growth	0.172	0.179	0.340		1.187			
**Ecological trap**				2713		47.71	95.43	<0.001
Resource use	−0.604	0.214	0.005		0.547			
Predation risk	0.243	0.217	0.260		1.275			
Resource use * Predation risk	0.181	0.178	0.310		1.198			
**Non-ideal resource use +** **Hiding cover**				2713		47.41	94.84	<0.001
Resource use	−0.509	0.189	0.007		0.601			
Predation risk	0.168	0.204	0.410		1.183			
Vegetation growth	−0.178	0.272	0.510		0.837			
**Resource use**	−0.608	0.229	0.008	2713	0.545	54.56	109.12	<0.001
**Non-ideal resource use**				2713		47.19	94.38	<0.001
Resource use	−0.497	0.189	0.008		0.608			
Predation risk	0.175	0.204	0.390		1.192			
**Predation risk**	0.270	0.194	0.160	2713	1.310	41.95	83.90	<0.001
**Winter severity index**	1.159	0.415	0.005	2713	3.187	44.37	88.75	<0.001
**Hiding cover**	−0.115	0.264	0.660	2713	0.892	40.28	80.58	0.002

Models included individual fawn and year as random effects on the intercept. Models presented with standardized parameter estimates, standard errors (SE), probability values, degrees of freedom (*df*), and estimated hazard ratio parameter probability values, and percent integrated deviance explained indicating the reduction in the log-likelihood from the null model. Percent deviance explained was used to rank models. Model fit was assessed using a Chi-square test of log-likelihood of a given model (Log-likelihood *X* ^2^) compared to the null model.

## Discussion

Daily survival of fawns was most explained by our predictions related to non-ideal resource use and maternal nutritional effects. The interaction among these factors provides evidence that neonatal white-tailed deer survival can be influenced by annual interactions among several biological and environmental factors, as in other ungulates [15], [37]. Support for non-ideal resource use suggested that dams placed fawns [44] in habitats with poor resources, but predation risk within these habitats led to additive mortalities beyond those related to poor resources [42]. However, poor resource use appeared to explain most of the variation in survival, as predation risk was not significant within any models incorporating predation risk. Nonetheless, the significant positive interaction of resource use and predation risk within the ecological trap and maternal effects model suggests that predation may have partially mediated resource selection of dams. Support for maternal effects suggested that greater winter severity preceding fawn parturition likely reduced maternal nutritional condition, which carried over to fawns having decreased body mass at birth. Nutritional carry-over effects are common in ungulates [82] and can predispose neonates to greater mortality risk, particularly predation [7]. Hence, use of poor resources may have been exacerbated for fawns born following more severe winters due to poorer birth body condition, which would have made them more susceptible to mortality [83].

Why would dams place fawns in poor resources where predation had an additive effect on mortality of fawns, when this strategy would be detrimental to survival of fawns? We suggest that there are two possibilities to explain dams raising fawns in habitats near roads and avoiding lowland, deciduous, and coniferous forests and wetlands. The first is that dams perceived lowland forest as risky areas [48], [84], [85] based on long-term knowledge of core wolf territories in these interior forests (Svoboda, N.J., unpublished data, [48]). Wolves are the primary predator of white-tailed deer in northern latitudes [47]; therefore representing the greatest direct threat to deer survival [63]. The second possibility is that dams chose vegetation that provided spring growth, which placated their nutritional needs during spring-summer, despite the greater predator detection of fawns [24]. Lowland and coniferous forests and wetland would have provided less favorable foraging for dams, and inadequate sunlight for thermoregulation of fawns during spring [26]. Hence, raising fawns in habitats near roads could have provided dams with refuge cover [34], [86] to primarily increase nutritional intake [10] during spring, but also decrease the probability of encountering wolves [84], [87], [88], [89]. These maternal behavioral trade-offs are common to ungulates, whereby parturient females improve fitness by allocating specific vegetation to refugia, foraging, and maternal fawn care, thereby increasing nutritional intake and probability of survival of neonates [8], [10], [27], [90], [91]. However, these behavioral trade-offs may have decreased the survival of fawns in our study through subsequent spatial overlap with alternative predators [34].

As was seen when examining resource use by fawns, coyotes avoided or were excluded from core wolf territories (Svoboda, N.J., unpublished data, [92]), which likely led to greater resource overlap and predation of fawns by coyotes. Coyotes and bobcats appeared to shift from a generalist resource selection pattern to selection of specific resources when they began to detect fawns as a pulsed food resource (Svoboda, N.J., unpublished data). While coyotes and bobcat appeared to increase use of a search image for fawns, they also have body masses that allow them to energetically profit from opportunistically searching for fawns, compared to wolves which have a body mass too great to make fawn searching energetically profitable [93]. Additionally, dams may have tolerated greater predation risk of coyotes because coyotes were easier to fend off compared to wolves [8]. Greater detection of fawns by bobcats possibly resulted in bobcats having the second greatest number of fawn predation events during the study. Similar scenarios have been documented with caribou calves (*Rangifer tarandus* [90]) and pronghorn fawns (*Antilocapra americana*; [93]), where avoidance of primary predators led to greater predation from alternative predator species. Although coyotes were consistently the main mortality source and predator across years, we suggest that the predominant coyote predation of fawns was an indirect result of dams avoiding lowland forests, which likely had less desirable forage during spring-summer and greater wolf use. Although dams may have lost fawns to coyotes in some years, attaining adequate forage and avoiding wolves was likely important to improving their lifetime reproductive success [31], particularly as females up to 15.5 years old were pregnant [94] and could have produced numerous generations of fawns.

Adequate hiding cover from predators can increase survival of neonatal ungulates [8], [10], but only explained about 16% of the variation in fawn survival. While we expected years with earlier vegetation growth to provide more hiding cover, it is possible that the relatively mild winters we observed provided similar hiding cover across years. Additionally, we estimated vegetation growth about 1 June, which may be too early to detect the annual variation in spring vegetation growth that may affect hiding fawns. Predator efficacy, particularly of coyotes and bobcats (Svoboda, N.J., unpublished data), in locating fawns could have increased if hiding cover was limited and fawns were more spatially predictable across years [14]. Although fawn survival was more related to fawn body mass at birth than hiding cover, similar birth body mass between 2010 and 2011 suggests that a more severe winter in 2011 likely reduced forage and fawn hiding cover the following spring.

Predation risk can directly [21] or indirectly [8], [10], [50], [95] influence ungulate survival through behavioral trade-offs between resource use and predation risk from single predators. However, our study demonstrates that survival of white-tailed deer fawns was influenced not only by dam trade-offs in resource selection and multi-species predation risk, but also that perceived risk associated with each predator can influence species-specific predation rates of fawns. We recommend identifying the species-specific risk of all major predators when investigating free-ranging ungulates in multi-predator landscapes. We recognize that our understanding of the relationship between survival of fawns and predation risk was constrained because our predation risk data was based on the probability of fawns encountering predators, rather than the probability of fawns actually being killed [24]. Also, an insufficient number of mortalities of radiomarked dams occurred across years to compare their survival to resource selection and predator avoidance strategies, which limited our interpretation of these behaviors related to fitness. Nonetheless, survival (70%) of radiomarked adult females across years was greater than that of fawns (Duquette, J.F., unpublished data) and supported our interpretations of dam resource selection and predator avoidance strategies to reduce mortality risk of dams. While poor forage across the study area and winter weather likely had maternal nutritional effects on fawns, predation risk also appeared to mediate resource use. Observed interactions among resource use, predation risk, and nutritional effects suggest that wildlife managers should emphasize practices that increase year-round forage quality and heterogeneity [13]. Using this habitat management regimen could help to increase fawn body mass at birth, reduce predation risk, and increase fawn survival during the period when they are maternally dependent.
